# Replacement of Mouse Microglia With Human Induced Pluripotent Stem Cell (hiPSC)-Derived Microglia in Mouse Organotypic Slice Cultures

**DOI:** 10.3389/fncel.2022.918442

**Published:** 2022-07-13

**Authors:** Ari Ogaki, Yuji Ikegaya, Ryuta Koyama

**Affiliations:** ^1^Laboratory of Chemical Pharmacology, Graduate School of Pharmaceutical Sciences, The University of Tokyo, Bunkyo, Japan; ^2^Institute for AI and Beyond, The University of Tokyo, Bunkyo, Japan; ^3^Center for Information and Neural Networks, National Institute of Information and Communications Technology, Suita, Japan

**Keywords:** microglia, transplantation, hiPSC, slice culture, phagocytosis

## Abstract

Microglia, the major immune cells in the brain, are reported to differ in gene expression patterns among species. Therefore, it would be preferable in some cases to use human microglia rather than mouse microglia in microglia-targeted disease research. In the past half a decade, researchers have developed *in vivo* transplantation methods in which human induced pluripotent stem cell-derived microglia (hiPSC-MG) are transplanted into a living mouse brain. However, *in vivo* transplantation methods are not necessarily accessible to all researchers due to the difficulty of obtaining the materials needed and the transplantation technique itself. In addition, for *in vivo* systems for microglia-targeted drug screening, it is difficult to control the pharmacokinetics, especially blood-brain barrier permeability. Therefore, in addition to existing *in vivo* transplantation systems, the development of an *ex vivo* transplantation system would help to further evaluate the properties of hiPSC-MG. In this study, we aimed to establish a method to efficiently transplant hiPSC-MG into cultured mouse hippocampal slices. We found that approximately 80% of the total microglia in a cultured slice were replaced by hiPSC-derived microglia when innate microglia were pharmacologically removed prior to transplantation. Furthermore, when neuronal death was induced by applying Kainic acid (KA) to slice cultures, transplanted hiPSC-MG changed their morphology and phagocytosed cell debris. Thus, this study provides a method to transplant hiPSC-MG into the mouse hippocampal slice cultures with a high replacement rate. Because the transplanted microglia survived and exerted phagocytic functions, this method will be useful for evaluating the properties of hiPSC-MG *ex vivo*.

## Introduction

In studies mainly of mouse microglia, researchers have shown that microglia play important roles in maintaining brain homeostasis, including the refinement of neural circuits as well as the removal of dead cells and cytokine release (Stevens et al., 2007; Neumann et al., 2009; Li and Barres, 2018; Liu et al., 2021). Recently, however, mouse and human microglia have been shown to exhibit differences in several gene expression patterns (Zhang et al., 2016; Galatro et al., 2017; Masuda et al., 2019). In particular, it has been reported that there are species differences between human and mouse microglia in the “sensome”, a group of genes involved in the detection of changes in the surrounding environment, which is the main function of surveying microglia (Abels et al., 2021). Therefore, it would be desirable to use human microglia, especially in the study of brain diseases targeting microglial function. For this purpose, microglia derived from human induced pluripotent stem cells (hiPSC-MG) have been developed and used given the advantages with regard to availability and ethics. Although there are minor methodological differences, hiPSC-MG are basically obtained by differentiating hiPSCs into hematopoietic progenitor cells (Abud et al., 2017; Douvaras et al., 2017; McQuade et al., 2018), mainly because microglia originate from myeloid progenitor cells in the yolk sac (Ginhoux et al., 2010; Kierdorf et al., 2013; Abud et al., 2017; Prinz et al., 2017; McQuade et al., 2018).

To study the function of hiPSC-MG *in vivo*, researchers have developed several methods to transplant hiPSC-MG into a living mouse brain (Abud et al., 2017; McQuade et al., 2018; Svoboda et al., 2019; Xu et al., 2020; Parajuli et al., 2021). In addition to the transplantation of hiPSC-MG, transplantation of human induced hematopoietic progenitor cells (hiHPCs), which are progenitor cells of microglia differentiated from hiPSCs, has also been developed (Hasselmann et al., 2019). Transplanted hiHPC-derived microglia (hiHPC-MG) in the mouse brain have been shown to maintain the gene expression patterns of human microglia, respond to laser ablation and lipopolysaccharide (Hasselmann et al., 2019), and phagocytose synapses (Xu et al., 2020). In a mouse model of Alzheimer’s disease, transplanted hiHPC-MG phagocytosed amyloid-β (Aβ; Abud et al., 2017; Hasselmann et al., 2019). Furthermore, genes highly expressed in hiHPC-MG around Aβ are different from those in mouse microglia around Aβ, showing that the hiHPC-MG transplantation methods are useful for validating human microglia-specific dynamics *in vivo* (Hasselmann et al., 2019).

However, *in vivo* transplantation systems have difficulties that must be considered when they are used for drug screening. For example, drug metabolism and delivery to the brain parenchyma across the blood-brain barrier must be considered. Above all, experimentally, it is difficult to increase the number of mice transplanted with a stable number of hiPSC-MG. The replacement rate of microglia among brain regions is highly variable, with 80% of microglia replaced by hiPSC-MG in the hippocampus, while almost no hiPSC-MG were present in the cortex 60 days after intranasal administration of hiPSC-MG (Parajuli et al., 2021). Furthermore, many *in vivo* transplantation models use immunodeficient mice, such as the MITRG, Rag2−/−, and NSG-Quad mouse lines, to avoid immune rejection of transplanted hiPSC-MG (Abud et al., 2017; McQuade et al., 2018; Hasselmann et al., 2019; Svoboda et al., 2019; Xu et al., 2020). The transgenic mouse line used for transplantation is also a factor that strongly affects the replacement rate. For example, Svoboda et al. found a 20%–30% replacement rate in the cortex after 2 months of hiPSC-MG administration to NSG-Quad mice, whereas Hasselmann et al. reported a 70%–80% replacement rate in MITRG mice in the same region and with the same timing after administration (Hasselmann et al., 2019; Svoboda et al., 2019).

Notably, immunodeficient mice lack genes involved in the immune system, which would also affect the entire immune system and likely disrupt the interaction between microglia and peripheral immune cells that modulate brain function (Dionisio-Santos et al., 2019; Bettcher et al., 2021). In addition, in some cases, these mice will be crossed with as many as three transgenic mouse lines expressing human macrophage colony stimulating factor (CSF), human interleukin (IL) 2 and 3, and human thrombopoietin to enhance the survival of hiPSC-MG. Thus, the experimenter must expend effort to maintain and breed multiple mouse strains.

In this study, we aimed to establish an *ex vivo* system to study the properties of transplanted hiPSC-MG. For this purpose, we transplanted hiPSC-MGinto a mouse cultured brain slice that maintains multiple types of brain cells, neural circuits, and extracellular matrix. We depleted innate mouse microglia in a cultured hippocampal slice prior to transplantation of hiPSC-MG by applying PLX3397, an inhibitor of colony stimulating factor 1 α receptor (CSF1-α R), or clodronate liposomes (Araki et al., 2020). We used commercially available iCell microglia (iCell-MG), which are differentiated from hiPSCs into microglia following the protocol established by Abud et al. (2017). Furthermore, we tested whether the transplanted hiPSC-MGs have phagocytosis ability, which is the main function of microglia. In this study, we induced neuronal cell death by kainic acid (KA) and examined the microglial phagocytosis. It has been reported that KA can induce both neuronal apoptosis and necrosis (Simonian et al., 1996; Fujikawa et al., 2000). In both processes of cell death, phosphatidylserine (PS), a phospholipid with a negatively charged head group, is externalized to the membrane surface, which is recognized by macrophages for subsequent phagocytosis (Fadok et al., 1998; Shlomovitz et al., 2019). Microglia also phagocytose cell debris and synapses by recognizing PS (Grommes et al., 2008; Graham et al., 2014; Shirotani et al., 2019; Scott-Hewitt et al., 2020). Therefore, we investigated the possibility that PS-mediated phagocytosis of dead cells may also occur in hiPSC-MG.

To our knowledge, the current study is the first report to compare the most efficient way to transplant hiPSC-MG into mouse brain slice cultures, and the method will contribute to the promotion of research targeting hiPSC-MG.

## Materials and Methods

### Animals

Experiments were performed with the approval of the animal experiment ethics committee at the University of Tokyo (approval number: P29–10) and according to the University of Tokyo’s guidelines for the care and use of the laboratory animals. Experiments were conducted using postnatal day 6 (P6) C57BL/6J mice. The mice were housed under a controlled temperature and light schedule (23–25°C and a 12-h light/dark cycle) and given unlimited access to food and water.

### Slice Culture

The preparation and maintenance of slice cultures, including culture media, were performed as previously described (Kasahara et al., 2016; Ogaki et al., 2020). To prepare slice cultures, P6 mouse brains were sectioned into 400-μm-thick horizontal slices using a DTK-1500 vibratome (Dosaka, Kyoto, Japan) in aerated, ice-cold Gey’s balanced salt solution (GBSS) containing 36 mM glucose. The entorhinohippocampal regions of slices were dissected out and incubated for 30–90 min at 4°C in an incubation medium containing minimal essential medium (MEM; M4655; Sigma, St. Louis, MO, USA), 9.0 mM Tris, 22.9 mM HEPES, and 63.1 mM glucose supplied with penicillin and streptomycin (#15140-122; Thermo Fisher, Waltham, MA, USA). Following this incubation, the slices were placed on PTFE membrane filters (JHWP02500; Merck Millipore, Billerica, MA, USA) on the doughnut plates (Hazai-Ya, Tokyo, Japan; Koyama et al., 2007) in a solution containing 50% MEM, 25% horse serum (26050-088; Gibco, Grand Island, NY, USA), 25% HBSS, 6.6 mM Tris, 16.9 mM HEPES, and 4.0 mM NaHCO_3_ supplemented with 29.8 mM glucose and 1% gentamicin sulfate solution (16672-04; Nacalai, Kyoto, Japan). Finally, the slices were cultured at 35°C in a humidified incubator with 5% CO_2_ and 95% air. The culture medium was changed twice a week.

### Innate Microglial Depletion

Innate microglia were depleted from cultured slices with PLX3397 (also known as pexidartinib, CS-4256; Monmouth Junction, NJ, USA) or the liposomal clodronate Clophosome-A (F70101C-A; FormuMax, Sunnyvale, CA, USA; Araki et al., 2020). PLX3397 was dissolved in DMSO at 100 mM as a stock solution and stored at -25°C. A stock solution of PLX3397 was added to culture media at 30 μM from 0 to 7 days *in vitro* (DIV). In the control group, the same amount of DMSO was added to the culture media. At 7 DIV, cultured slices were carefully rinsed three times with warmed PBS, and fresh culture medium was added.

Liposomal clodronate was added to culture media at 0.05 mg/ml from 3 to 7 DIV. In the control group, the same amount of control liposomes was added to the culture media. At 7 DIV, cultured slices were carefully rinsed three times with warmed PBS, and a fresh culture medium was added.

### Kainic Acid (KA) Treatment

KA (0222; Tocris, Bristol, UK) was added to the culture media at 20 μm and treated for 24 h from 20 DIV. After KA treatment, cultured slices were fixed.

### Transplantation of iPSC-Derived Microglia

iPSC-derived iCell-MG (01279; FUJIFILM Wako Pure Chemical Co., Osaka, Japan; e-mail: fcdi-support@fujifilm.com; website address: https://www.fujifilmcdi.com/) were purchased and stored in liquid nitrogen before seeding. iCell-MG were generated based on the protocol from Abud et al. (2017). At 10 DIV, iCell-MG was seeded onto cultured slices. At 10 DIV, iCell-MG were seeded onto cultured slices. iCell-MG were dissolved for 3 min at 37°C, suspended in 10 ml of culture media, and gently pipetted 2–3 times before counting. The number of viable iCell-MG were assessed using trypan blue stain solution (29853-34, Nacalai, Kyoto, Tokyo) that labels dead cells. The number of viable iCell-MG was 2–5 × 10^6^ cells per vial. After that, iCell-MG were centrifuged at 1,000×*g* for 10 min at room temperature. Since most primary cells cannot tolerate 1,000×*g* force when being spun down, those methods may not be appropriate for iCell-MG preparation, however, we followed according to the user’s guide. After centrifugation, the supernatant was discarded and resuspended in 2 μl per slice of culture media with 0.5 μl per slice of recombinant human macrophage CSF (human M-CSF also known as CSF1; 133-13611; FujifilmWako Pure Chemical Co., Osaka, Japan). Human M-CSF was dissolved in PBS at 10 mM and stored at -25°C. iCell-MG were seeded at 1.0 × 10^5^ cells per slice gently with a P2 pipette onto cultured slices. After seeding, cultured slices were treated with human M-CSF at 10 μM twice a week with medium change.

### Immunohistochemistry and PSVue Staining

**Figure 1:**
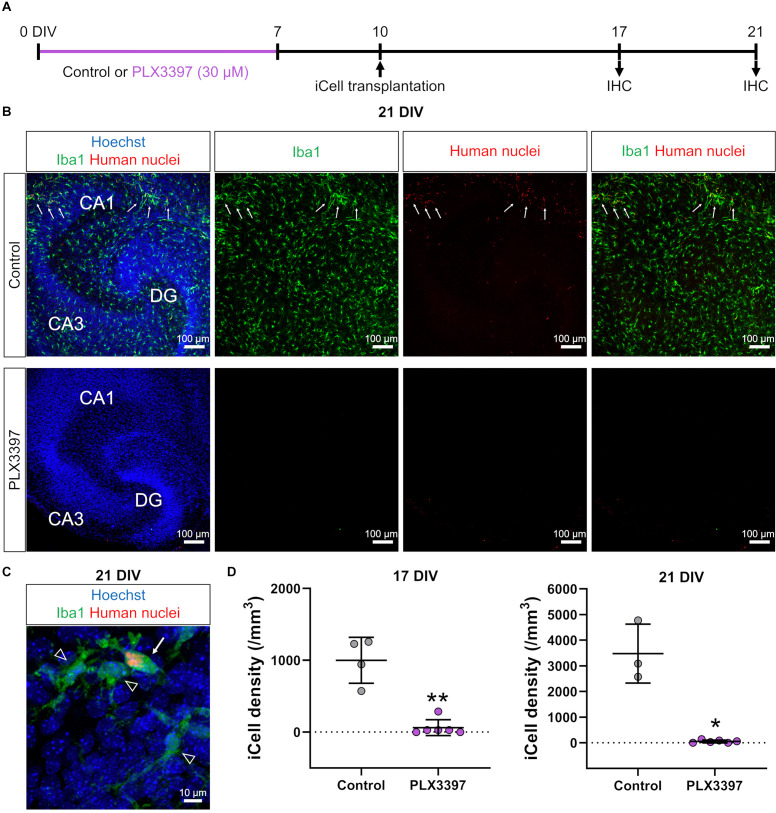
PLX3397 attenuated the transplantation of iCell-MG in slice cultures. **(A)** Experimental paradigm. IHC: immunohistochemistry. **(B)** Representative images of iCell-MG-transplanted slices at 21 DIV cultured with (lower) or without (control, upper) 30 μM PLX3397. Cultures were immunostained for Iba1 (green) and human nuclei (red). Nuclei were stained with Hoechst (blue). Some iCell-MG were found in control cultures (upper panels, arrows). **(C)** Representative images of iCell-MG (arrow) and innate microglia (arrowheads) in control cultures. **(D)** iCell-MG density at 17 (left panel) and 21 DIV (right panel). In PLX3397, iCell density was zero in two of the six slices at 17 DIV and 21 DIV. **p < 0.01 and *p < 0.05, Mann-Whitney rank sum test, *n* = 4 (17 DIV in control), six (17 DIV in PLX3397), three (21 DIV in PLX3397), and six (21 DIVin PLX3397) slices, each from three mice. Data represent the mean ± SD.

**Figure 2:**
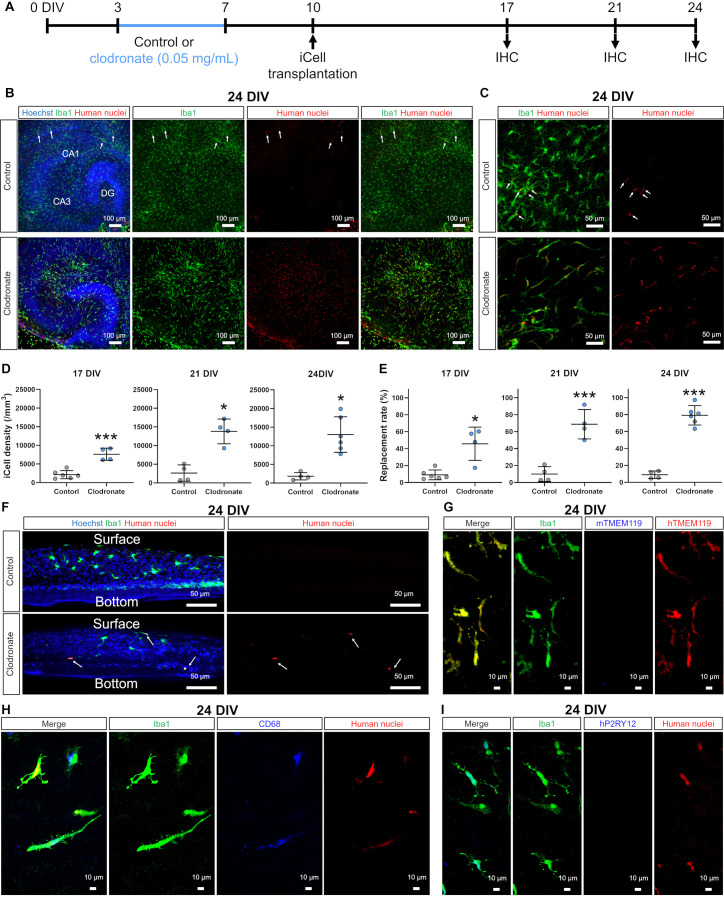
Clodronate promoted the transplantation of iCell-MG in slice cultures. **(A)** Experimental paradigm. **(B)** Representative images of iCell-MG-transplanted slices at 24 DIV cultured with (lower) and without clodronate (upper, control). Cultures were immunostained for Iba1 (green) and human nuclei (red). Nuclei were stained with Hoechst (blue). **(C)** Magnified images of control (upper panels) and clodronate-treated slices (lower panels) at 24 DIV. **(D)** iCell-MG density at 17 (left panel), 21 (middle panel), and 24 DIV (right panel). ***p < 0.001 and*p < 0.05, Student’s *t*-test, *n* = 6 (17 DIV in control), four (17 DIV in clodronate), four (21 DIV in control), four (21 DIV in clodronate), four (24 DIV in control), and six slices (24 DIV in clodronate), each from two mice. Data represent the mean ± SD. **(E)** Replacement rate of iCell-MG at 17 (left panel), 21 (middle panel), and 24 DIV (right panel). *p < 0.05 and***p < 0.001, Student’s *t*-test, *n* = 4–6 slices. Data represent the mean ± SD. **(F)** Representative images of innate and iCell microglial distribution at 24 DIV cultured with (lower) and without clodronate (upper, control). iCell-MG were distributed from the surface to near the bottom of slices (arrows). **(G)** Representative images of innate and iCell microglia at 24 DIV with clodronate. Cultures were immunostained for Iba1(green), mouse TMEM119 (mTMEM119; blue), and human TMEM119 (hTMEM119; red). **(H)** Representative images of innate and iCell microglia at 24 DIV with clodronate. Cultures were immunostained for Iba1 (green), CD68 (blue), and human nuclei (red). **(I)** Representative images of innate and iCell microglia at 24 DIV with clodronate. Cultures were immunostained for Iba1 (green), human P2RY12 (hP2RY12; blue), and human nuclei (red).

**Figure 3:**
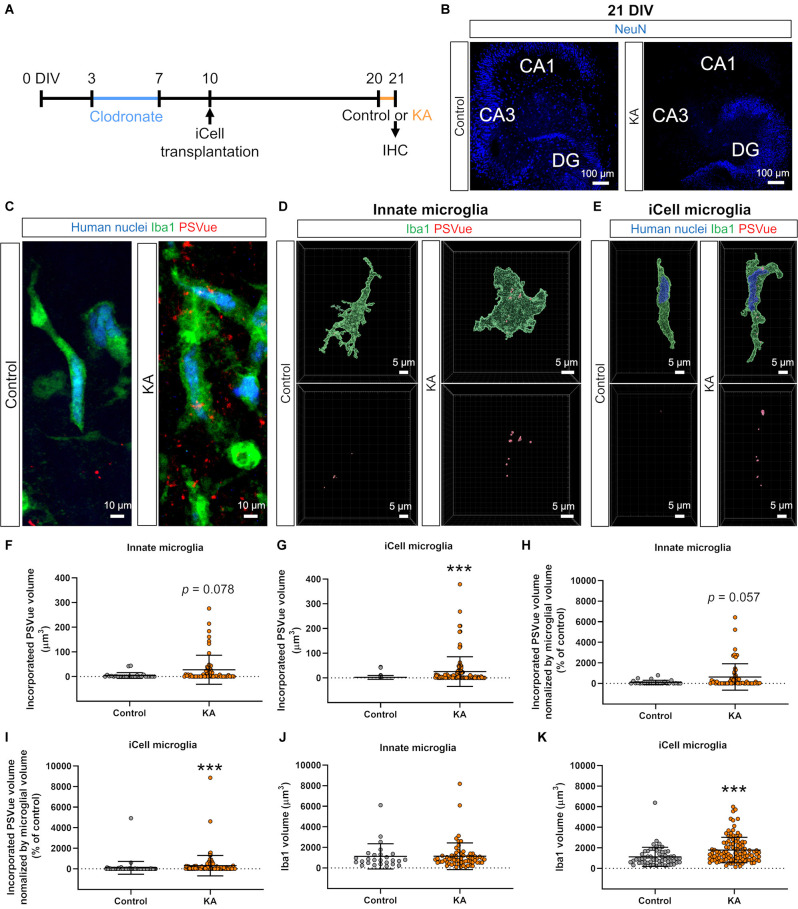
Transplanted iCell-MG phagocytosed phosphatidylserine (PS). **(A)** Experimental paradigm. **(B)** Representative images of cultures immunostained for NeuN at 21 DIV (blue). Neuronal cell death was induced in CA3 and CA1 fields with kainic acid (KA). **(C)** Representative images of iCell-MG in the control (left) and KA groups (right) at 21 DIV. Cultures were immunostained with human nuclei (blue) and Iba1 (green) following PSVue staining (red). **(D,E)** Three-dimensional (3D) reconstruction of innate microglia **(D)** and iCell-MG **(E)** that incorporated PSVue in the control and KA-treated cultures. **(F,G)** Incorporated PSVue volume per microglia by innate microglia **(F)** and iCell-MG **(G)**. ***p < 0.001, Mann-Whitney rank sum test, *n* = 26 (innate microglia in control), 58 (innate microglia in KA), 63 (iCell-MG in control), and 107 (iCell-MG in KA) cells, from six (control) and 10 (KA) slices, from four (control and KA) mice. Data represent the mean ± SD. **(H,I)** Incorporated PS volume per microglial volume by innate microglia **(H)** and iCell-MG **(I)**. ***p < 0.001, Mann-Whitney rank sum test, *n* = 26 (innate microglia in control), 58 (innate microglia in KA), 63 (iCell-MG in control), and 107 (iCell-MG in KA) cells, from six (control) and 10 (KA) slices, from four (control and KA) mice. **(J,K)** Innate microglial volume **(J)** and iCell-MG **(K)**. ***p < 0.001, Mann-Whitney rank sum test, *n* = 26 (innate microglia in control), 58 (innate microglia in KA), 63 (iCell-MG in control), and 107 (iCell-MG in KA) cells, from six (control) and 10 (KA) slices, from four (control and KA) mice. Data represent the mean ± SD.

**Figure 4:**
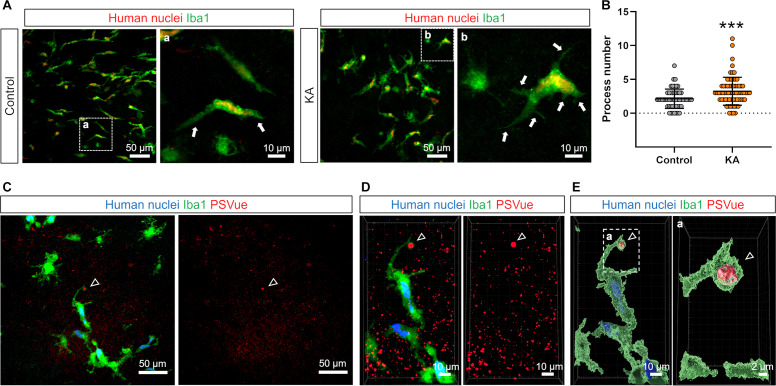
Transplanted iCell-MG formed phagocytic cups. **(A)** Representative images of iCell-MG in the control (upper) and KA (lower) slice cultures immunostained for human nuclei (red) and Iba1 (green) at 21 DIV. Magnified images of the regions outlined by dashed squares are shown (right panels, in **A** and **B**). iCell-MG had more processes in the KA-treated cultures than the control cultures (arrows). **(B)** The number of processes of iCell-MG (per cell) in the control and KA-treated cultures. The process number was increased by KA treatment.***p < 0.001, Mann-Whitney rank sum test, *n* = 53 and 69 cells from two slices, each from one mouse. Data represent the mean ± SD. **(C)** Representative images of iCell-MG in the KA-treated cultures immunostained for human nuclei (blue) and Iba1 (green) following PSVue staining (red). PS was encapsulated in an Iba1-positive phagocytic cup (arrowheads). **(D,E)** A three-dimensional (3D) reconstruction of the images shown in **(D)**. PS was encapsulated in an Iba1-positive phagocytic cup (**D,E**, arrowheads). Magnified images of the regions outlined by dashed squares are shown (right panel, in **A–E**).

Cultured slices were fixed in 4% paraformaldehyde at 4°C for 24 h. For PSVue and Hoechst staining, the samples were subsequently incubated with PSVue^®^ 643 (1:100; P-1006; Molecular Targeting Technologies, West Chester, PA) and Hoechst 33342 (1:500; Thermo Fisher, Waltham, MA, USA) in PBS overnight at room temperature with agitation. Next, the slices were permeabilized and blocked for 1 h using 0.3% Triton X-100 with 10% goat serum in PBS. Primary antibody staining was performed using mouse anti-NeuN (1:1,000; MAB377; Merck Millipore, Burlington, MA, USA), rabbit anti-Iba1 (1:1,000; 019-19741; FujifilmWako Pure Chemical Co., Osaka, Japan), mouse anti-human nuclei (MAB1281; Millipore, Bedford, MA, USA), rabbit anti-P2RY12 (1:500; ab183066; Abcam, Cambridge, UK), rabbit anti-human TMEM119 (1:500; Thermo Fisher, Waltham, MA, USA), mouse anti-mouse TMEM119 (1:500; 400011; Synaptic Systems, Göttingen, Germany), and rat anti-CD68 (1:500; MCA1957GA; Bio-Rad, CA, USA) followed by Alexa Fluor 488-, 594-, and 647-conjugated secondary antibody staining (1:500; Thermo Fisher, Waltham, MA, USA). Finally, the samples were embedded in Permafluor (Thermo Fisher, Waltham, MA, USA). Images of immunostained samples were obtained using the SpinSR10 (Olympus, Tokyo, Japan) confocal system with 10× (NA = 0.40), 20× (NA = 0.75), and 40× (NA = 0.95) objectives. In iCell-MG distribution analysis, slices on the membranes were cut vertically after immunostaining (Kasahara et al., 2016). Z-series images were collected at 2.0 μm steps and stacked for 11 slices for iCell-MG density and NeuN analysis (Figures 1, 2, 3B), 0.5 μm steps for eight slices for iCell-MG distribution analysis (Figure 2F), 0.33 μm steps for 31 slices for microglial engulfment analysis (Figures 3C–I) and stacked for microglial marker expression (Figures 2G–I) and microglial morphological analysis (Figure 4). The stacked images were analyzed using ImageJ software (NIH, Bethesda, MD, USA). The number of the innate and iCell-MG and iCell-MG processes were counted manually. The volume of incorporated PSVue and Iba1 were analyzed with Sync Measure 3D in ImageJ after image thresholding. Imaris software (Carl Zeiss Vision GmbH, Aalen, Germany) was used for preparing microglial three-dimensional (3D) reconstruction images with surface creation mode (Figures 3D,E, 4D,E).

### Statistical Analysis

Data were subjected to parametric or nonparametric tests, depending on the results of the test of normality. Data were presented as the mean ± standard deviation (SD). Data were statistically analyzed by researchers blinded to experimental conditions. Statistical analyses used can be found in each figure legends: Mann-Whitney rank sum test was performed for iCell density in PLX3397 in Figure 1D, for incorporated PSVue volume, incorporated PSVue volume normalized by microglial volume, and Iba1 volume in Figures 3F–K, and for process number in Figure 4; Student’s *t*-test was performed for iCell density and transplantation rate in clodronate in Figures 2D,E.

## Results

First, we treated brain slice cultures with PLX3397, an antagonist of the CSF1 receptor (CSF1R), which is expressed in microglia and involved in microglial survival. PLX3397 is widely used to remove microglia and macrophages, and we previously reported that 30 μM PLX3397 efficiently removes microglia from brain slice cultures (Araki et al., 2020). In the current study, PLX3397 (30 μM) was administered from 0 to 7 DIV to remove innate microglia (Figure 1A). Then, to transplant iCell-MG in slice cultures, we drop-applied iCell-MG onto the surface of cultured slices at 10 DIV after a 3-day recovery period because the remaining PLX3397 could affect the survival of iCell-MG as well as the innate microglia expressing CSF1R. To increase the viability of iCell-MG by activating CSF1R, we added human M-CSF constantly to the culture medium with medium changes (twice a week) from 10 DIV until fixation (Elmore et al., 2014).

We fixed slice cultures at 17 or 21 DIV and immunostained the cultures (Figure 1A) with antibodies against Iba1 to detect microglia and against human nuclei to detect human-derived cells, i.e., iCell-MG, which allowed us to separate innate microglia (Iba1-positive and human nuclei-negative; Figure 1C, arrowheads) from iCell-MG (Iba1-positive and human nuclei-positive; Figure 1C, arrow). We found that innate mouse microglia dominated slice cultures at 21 DIV (Figure 1B). We also found some human nuclei-immunopositive iCell-MG (Figure 1B, arrows), and the percentage of iCell-MG among the total microglia was 12.3 ± 2.5%. In the PLX3397-treated slices, neither innate microglia nor iCell-MG was found (Figure 1B), suggesting the continued cytotoxic effect of PLX3397 even after the recovery period from 7 to 10 DIV. The same trend was observed at both 17 DIV and 21 DIV, where the density of iCell-MG was lower in the PLX3397 group than in the control group (Figure 1D).

Because PLX3397 prevented efficient transplantation of iCell-MG (Figure 1), we next removed innate microglia by applying clodronate liposomes (0.05 mg/ml), which are known to effectively deplete mouse splenic macrophages and can also remove microglia in brain slice cultures (Kameka et al., 2014; Araki et al., 2020). After treatment with clodronate liposomes from 3 to 7 DIV, iCell-MG was drop-applied onto cultured slices at 10 DIV with a 3-day recovery period to minimize the effect of residual clodronate on iCell-MG (Figure 2A). At 24 DIV, most of the microglia in the control cultures were innate microglia, and some were iCell-MG (arrows in Figures 2B,C control), which is similar to the case of PLX3397 (Figure 1B control, arrows). In the clodronate-treated slices, 20.8 ± 10.5% of total microglia were innate microglia, and 79.2 ± 10.5% were replaced by iCell-MG (Figures 2B–clodronate,E), which is different from the case of PLX3397 treatment (Figures 1B–D). The density of iCell-MG was higher in the clodronate group than in the control group throughout the culture period (17, 21, and 24 DIV; Figure 2D). In addition, the replacement rate, which was calculated as the number of iCell-MG divided by total microglia in each slice, gradually increased from 17 to 24 DIV, and on average, approximately 80% of the total microglia were replaced by iCell-MG at 24 DIV (Figure 2E). Transplanted iCell-MG were distributed from the surface of cultured slices to near the bottom (Figure 2F). In addition, all the transplanted iCell-MG were immunopositive for TMEM119 (Figure 2G) and some of them were immunopositive for CD68 (Figure 2H). Additionally, no iCell-MG was immunopositive for P2RY12 (Figure 2I). Based on data from the manufacturer, more than 80% of iCell-MG expressed P2RY12 and TMEM119 (no data for CD68). One of the possible reasons for these differences is that transplantation of iCell-MG into mouse slice cultures and 14 days of culture may have altered their gene expression and translation into protein.

In research of transplanted hiPSC-MG for the treatment of brain diseases, especially neurodegenerative diseases, one major objective will be to examine the phagocytic function of hiPSC-MG because the removal of dead cells, cell debris, and endogenous substances in the brain, such as Aβ, can be a therapeutic strategy. Thus, we examined whether transplanted iCell-MG have a phagocytic function in clodronate-treated slice cultures (Figure 3A). To efficiently test phagocytosis by iCell-MG, we induced neuronal cell death by kainic acid (KA) treatment. KA is a kainate receptor agonist that induces hippocampal neuronal loss, especially pyramidal neurons in CA1 and CA3 fields, both *in vivo* and in slice cultures (Araki et al., 2020), and we confirmed that KA treatment at 20 DIV for 24 h (Figure 3A) resulted in a decrease in neuronal nuclear marker NeuN-positive cells both in the CA1 and CA3 fields (Figure 3B).

We visualized PS on the membrane surface by using PSVue, a fluorescent dye with a dinuclear zinc complex, prior to permeabilization for further immunostaining with Iba1 and human nuclei antibodies (Figure 3C). After KA treatment, externalized PS was increased in the CA3 fields, where neuronal cell death widely occurred (Figure 3C). We then examined microglial phagocytosis of PS-externalized cell debris by measuring the PSVue volumes in microglia. We selected cells that were not in contact with other microglia or iCell-MG as the target cells for quantification, in order to accurately quantify the number of processes and volumes. We chose the hippocampal CA3 region for quantification because it is the region where KA-induced neuronal cell death occurs extensively and the associated appearance of PSVue is detected. Three-dimensional reconstruction of immunostained microglia in slice cultures revealed that both innate microglia and transplanted iCell-MG incorporated PS (Figures 3D,E), indicating the phagocytosis of PS-labeled debris by these cells. In addition, the incorporated PS signal increased in iCell-MG in the KA group compared to the control group, while there was a tendency to increase in innate microglia (*p* = 0.078; Figures 3D,E). The volume of incorporated PS per cell was increased by KA treatment in both innate microglia (*p* = 0.057) and iCell-MG (*p* < 0.001; Figures 3F,G). To normalize the volume of each microglia, we also quantified the amount of phagocytosis of PS divided by the volume of cells, confirming that KA treatment increased PS phagocytosis both in innate microglia and in iCell-MG (Figures 3H,I). The percentage of phagocytic microglia, which were defined as microglia that incorporated PSVue volume over 0 μm^3^, was 61.5% in control and 69.0% in KA in innate microglia (Figure 3F), and 55.6% in control and 86.0% in KA in iCell-MG (Figure 3G). Iba1 volume of iCell-MG was increased by KA treatment, while that of innate microglia was not varied (Figures 3J,K). The innate microglia in this context are those that survived (or proliferated) after clodronate treatment (note that iCell-MG were not treated with clodronate), and the possible reason why innate microglia are less responsive is that these innate microglia changed their property and are no longer naïve in terms of the response to KA treatment.

It was also reported that KA treatment affects microglial morphology. Eyo et al. showed that intracerebroventricular administration of KA increases the number of microglial primary processes through the activation of P2Y12 receptors in microglia, which are induced by adenosine triphosphate (ATP) released by neuronal overexcitation (Eyo et al., 2014). Thus, we finally investigated whether transplanted iCell-MG also exhibited morphological changes after KA treatment in the CA3 field of cultured slices (Figure 4A). We found that most iCell-MG in the control had a bipolar morphology with a few processes, whereas iCell-MG possessed multiple processes (Figure 4B).

During phagocytosis of cellular debris, microglia sometimes form phagocytic cups on the tips of their processes through actin polymerization and associated cytoskeletal remodeling (Lee et al., 2007; Sierra et al., 2013). Thus, we finally examined whether transplanted iCell-MG can form phagocytic cups to confirm their phagocytic capacity. When neuronal cell death was induced by KA treatment, iCell-MGwith a phagocytic cup was present, and PS was encapsulated in the phagocytic cups (Figures 4C–E). In control, 0% of innate microglia and 6.3 ± 12.5% of iCell-MG had phagocytic cups in CA3 (*n* = 4 mice). In KA-treated cultures, 10.7 ± 11.1% of innate microglia and 3.1 ± 2.8% of iCell-MG had phagocytic cups in CA3 (*n* = 5 mice). Since the phagocytic cup is an essential structure specific for phagocytosis by microglia, our findings suggest that iCell-MG maintain phagocytic function. However, some reports suggested that the treatment of KA in the primary culture of microglia and BV-2 microglia cell line changes microglial cell morphology and cytokine release (Zheng et al., 2010; Li et al., 2020). Therefore, we cannot rule out the possibility that the direct effects of KA on microglia are responsible for the changes in microglial morphology such as process number, cell volume, and phagocytic cup formation caused by KA in this study.

## Discussion

To examine the interaction between hiPSC-MG and other cell types as therapeutic targets for brain diseases, we had to establish a user-friendly drug screening system. For this purpose, we developed a method to efficiently transplant iCell-MG into a mouse-derived brain slice culture system to replace innate mouse microglia.

When innate microglia were removed by PLX3397 in slice cultures, transplanted iCell-MG barely survived in slice cultures. However, when innate microglia were removed by clodronate, iCell-MG efficiently replaced innate microglia, and approximately 80% of the total microglia in slice cultures were iCell-MG 2 weeks after transplantation (24 DIV). Based on the report by Khoshnan et al. that PLX3397 at 0.3 and 3 μM did not efficiently remove microglia in rat brain slice cultures and that PLX3397 at 30 μM was most effective (Khoshnan et al., 2017), microglia were removed with 30 μM in this study. However, the possibility that 30 μM PLX was cytotoxic cannot be ruled out. Although it is necessary to consider the different doses and time courses in each report, the removal rate of microglia in mice was reported to be over 90% with PLX3397 compared to 70%–80% with clodronate (Han et al., 2017). Thus, PLX3397 is likely more cytotoxic to microglia than clodronate and probably reduced the viability of the implanted iCell-MGin cultured slices. It should also be noted that while some reports indicate that the process of selective removal of microglia by clodronate induces astrocyte activation (Han et al., 2019), others report that astrocytes are not affected (Kumamaru et al., 2012). In this study, we did not examine whether astrocytes are affected by the removal of microglia by clodronate. The replacement rate of hiPSC-MG has been reported to range from an average of 20% to 80% *in vivo*, depending on the experimental time courses and transplantation methods. In addition, the replacement rate significantly varies across brain regions *in vivo* (Hasselmann et al., 2019; Svoboda et al., 2019; Xu et al., 2020), mainly due to high variability across brain regions in the removal rate of innate microglia (Elmore et al., 2015; Spangenberg et al., 2016; Nelson and Lenz, 2017). Notably, each process of *in vivo* transplantation requires finely controlled and mature techniques, such as stable anesthesia, head fixation, and cannula insertion, to minimize possible brain damage from the transplantation process itself. These issues may be responsible for the variability in replacement rates.

In the current study, iCell-MG did not reproduce the ramified morphology of innate microglia and showed bipolar morphology. This finding may be due to the low expression of genes related to the elongation and branching of processes in iCell-MG. Although this finding applies to hiPSC-MG in general, including iCell-MG, it will be necessary to comprehensively examine the expressed genes, such as phagocytosis- and surveillance-related genes, depending on the purpose of the research. In addition, considering the application of hiPSC-MG to drug screening systems, it is necessary to examine the reproducibility of functional features of microglia, such as accumulation at inflammatory sites and synaptic phagocytosis in target diseases.

Finally, the method of hiPSC-MG transplantation in slice culture reported here is a simple method that can be directly used to study the interaction between hiPSC-MG and other brain cell types and is expected to contribute to drug discovery screening for diseases in the future. For this purpose, the CRISPR/Cas9 system can be used to genetically modify hiPSC-MG to target the microglia-specific causative genes of each brain disease. For example, Lin et al. utilized the CRISPR/Cas9 system to express APOE4 in hiPSCs, differentiating them into microglia-like cells (Lin et al., 2018). With this approach, it will be possible to manipulate disease-associated microglial genes in iPSC-MG, such as mutated C-X3-C motif chemokine receptor 1 (CX3CR1) in schizophrenia and autism spectrum disorder (Ishizuka et al., 2017) and mutated CSF1R in hereditary diffuse leukoencephalopathy with spheroid (HDLS) (Nicholson et al., 2013).

In this study, we developed an efficient method to transplant hiPSC-MG into mouse slice cultures, in which innate microglia were removed by clodronate in advance. We also found that transplanted hiPSC-MG have phagocytic ability. Our method is a simple system using commercially available hiPSC-MG and clodronate, and will be useful for studies that comprehensively examine the function of human microglia in neural circuits, including drug screening.

## Data Availability Statement

The raw data supporting the conclusions of this article will be made available by the authors, without undue reservation.

## Ethics Statement

The animal study was reviewed and approved by The animal experiment ethics committee at the University of Tokyo (approval number: P29-10).

## Author Contributions

AO conducted the experiments, analyzed the experimental data, and wrote the manuscript. YI discussed the results and commented on the manuscript. RK designed and planned the project and wrote the manuscript. All authors contributed to the article and approved the submitted version.

## Conflict of Interest

RK was financially supported by Takeda Pharmaceutical Company, Limited (Tokyo, Japan). The remaining authors declare that the research was conducted in the absence of any commercial or financial relationships that could be construed as a potential conflict of interest.

## Publisher’s Note

All claims expressed in this article are solely those of the authors and do not necessarily represent those of their affiliated organizations, or those of the publisher, the editors and the reviewers. Any product that may be evaluated in this article, or claim that may be made by its manufacturer, is not guaranteed or endorsed by the publisher.
